# Heat acclimation ameliorated heat stress-induced acute kidney injury and prevented changes in kidney macrophages and fibrosis

**DOI:** 10.1152/ajprenal.00065.2022

**Published:** 2022-07-07

**Authors:** Hiroyasu Goto, Masahiro Nakashima, Hiroyuki Nakashima, Midori Noguchi, Toshihiko Imakiire, Naoki Oshima, Manabu Kinoshita, Hiroo Kumagai

**Affiliations:** ^1^Department of Nephrology and Endocrinology, National Defense Medical College, Tokorozawa, Japan; ^2^Department of Immunology and Microbiology, National Defense Medical College, Tokorozawa, Japan

**Keywords:** acute kidney injury, heat acclimation, heat shock protein 70, heat stress, resident macrophages

## Abstract

Heatstroke can cause acute kidney injury (AKI), which reportedly progresses to chronic kidney disease. Kidney macrophages may be involved in such injury. Although heat acclimation (HA) provides thermal resilience, its renoprotective effect and mechanism remain unclear. To investigate heat stress-induced kidney injuries in mice and the mitigating effect of HA on them, male C57/BL6J mice were exposed to heat stress (40°C, 1 h) with or without 5-day HA (38°C, 3 h/day) prior to heat stress. Heat stress damaged kidney proximal tubules with an elevation of urinary kidney injury molecule-1. Kidney fibrosis was observed on *day 7* and correlated with urinary kidney injury molecule-1 levels on *day 3*. Kidney resident macrophages decreased on *day 1*, whereas the number of infiltrating macrophages in the kidney did not change. Both subsets of macrophages polarized to the proinflammatory M1 phenotype on *day 1*; however, they polarized to the anti-inflammatory M2 phenotype on *day 7*. HA significantly ameliorated heat stress-induced proximal tubular damage and kidney fibrosis. HA substantially increased heat shock protein 70 expression in the tubules before heat stress and reduced the elevation of cleaved caspase-3 expression after heat stress. HA also induced heat shock protein 70 expression of resident macrophages and prevented heat stress-induced changes in both subsets of kidney macrophages. These results provide pathophysiological data supporting the renoprotective effect of HA. Further studies are needed to confirm that HA can prevent kidney damage due to heat stress in humans.

**NEW & NOTEWORTHY** Heat stress could induce acute kidney injury. Although heat acclimation (HA) reportedly provides thermal tolerance, its effect on heat stress-induced kidney damage remains unclear. This study showed that 5-day HA ameliorates mouse kidney tubular damage and subsequent fibrosis caused by heat stress. It also demonstrated that HA enhances intracellular heat shock protein 70 expression in tubular cells and prevents a decrease in kidney resident macrophages, which explains the renoprotective effect of HA.

## Graphical abstract

**Figure:**
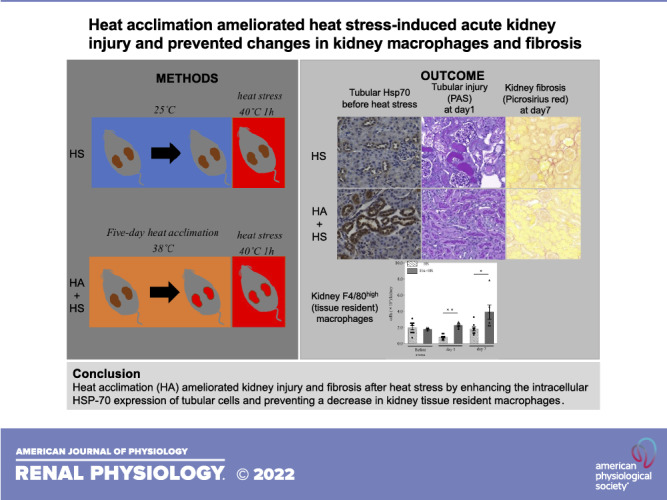

## INTRODUCTION

Recent global warming has caused an increase in the incidence of heat-related illnesses (1, 2). Among these illnesses, heatstroke, which is characterized by hyperthermia and multiorgan dysfunction, is the most hazardous and life-threatening condition (3). Exertional heatstroke occurs in association with physical exertion, and studies have investigated the condition in athletes, firefighters, and military personnel (4–6). Exertional heatstroke most often affects healthy young individuals, who sometimes develop acute kidney injury (AKI) (3, 7). Large cohort studies of military personnel have reported that ∼30–40% of patients with exertional heatstroke developed AKI (8, 9). In addition, Chapman et al. (10) recently compared subjects who exercised in a hot environment (around 40°C) with or without cooling their upper bodies and suggested that even mild hyperthermia (38.6 ± 0.4°C), which did not seem to induce heatstroke, can cause kidney damage. Furthermore, recent studies have suggested that such heat stress (HS)-induced AKI can induce chronic kidney disease (CKD), although the precise mechanism remains to be elucidated (4, 11).

Heat acclimation (HA) reduces the risk of heat related-illness and provides thermal tolerance, which is associated with an elevation of heat shock proteins (Hsps), and submaximal exercise performance (12–14). Intracellular expression of Hsps in human peripheral blood mononuclear cells was reportedly increased after HA, indicating cellular stability/resilience to HS (14). Nevertheless, it remains controversial as to whether HA reduces the risk of HS-induced AKI (15–17).

The pathogenesis of HS-induced AKI involves several mechanisms, including kidney hypoperfusion, rhabdomyolysis, and thermal injury (1). Recently, increasing evidence supports that kidney immune cells play important roles in early tissue injury, repair, and fibrosis after AKI (18–23). However, there have been few studies regarding the kidney immune responses to HS, in particular focusing on the effect of HA on the HS-associated immune response in the kidneys.

Mononuclear phagocytes, such as macrophages, monocytes, and dendritic cells, are important immune sentinels that maintain organ homeostasis and immunity in the kidney (24, 25). Circulating monocytes are recruited to inflamed tissues and differentiate into monocyte-derived macrophages. In contrast, tissue-resident macrophages have important functions in development, tissue homeostasis, and the resolution of inflammation (25). We and other researchers have reported that macrophages can be classified based on differential expression of F4/80 and CD11b into F4/80^high^ CD11b^low^ and F4/80^low^ CD11b^high^ subsets, which phenotypically resemble tissue-resident macrophages and infiltrative monocyte-derived macrophages, respectively (22, 24, 26–28). However, little is known about the role of macrophages in the kidney after exposure to HS.

In the present study, we investigated the effect of HS on kidney function, injury biomarker levels, and kidney pathology, including inflammation and fibrosis. We also examined changes in kidney macrophages after HS, focusing on tissue-resident and monocyte-derived macrophages. Finally, we examined whether HA can mitigate HS-induced kidney damage.

## MATERIALS AND METHODS

### Animals

Male C57/BL6J mice (age: 8–16 wk) were purchased from CLEA Japan (Tokyo, Japan). Mice were housed in a pathogen-free environment in cages maintained at 25°C and 40% relative humidity with a 12:12-h light-dark cycle. Experimental procedures were approved by the Ethics Committee of Animal Care and Experimentation of National Defense Medical College, Japan (Approval No. 20014).

### HS Protocol

**Figure 1.**
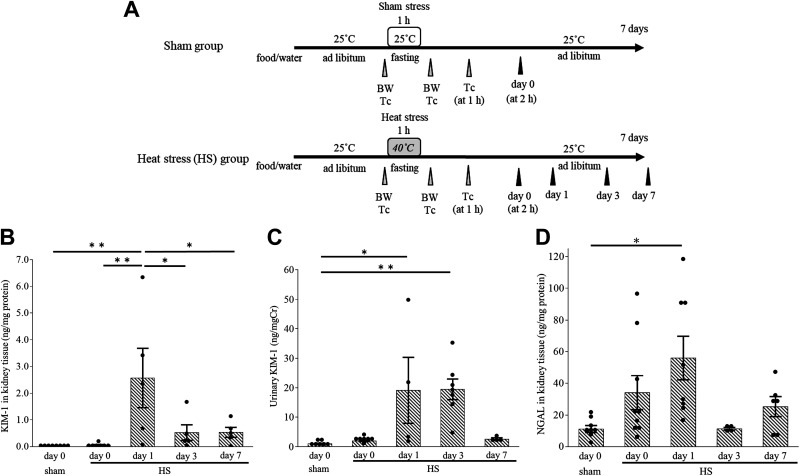
Mouse heat stress (HS) protocol and the evaluation of acute kidney injury after HS. *A*: mouse HS protocol. Mice were exposed to HS at 40°C and 40% relative humidity for 1 h on *day 0* (HS group). Samples were harvested on *days 0*, *1*, *3*, and *7*. Kidney injury molecule-1 (KIM-1) levels in kidney tissue (*B*) and urine (*C*) and neutrophil gelatinase-associated lipocalin (NGAL) levels in kidney tissue (*D*) were examined in HS and sham groups. The KIM-1 level in kidney extract was elevated on *day 1* (*B*) and that in urine was elevated on *days 1* and *3* (*C*). The kidney tissue NGAL level was elevated on *day 1* (*D*). Dots are actual data. Data represent means ± SE from 4 to 9 mice in each group. *P < 0.05; **P < 0.01. BW, body weight; T_C_, core temperature.

Mice were placed in an environmental chamber (STC-V, SANPLATEC, Osaka, Japan) at 40°C and 40% relative humidity for 1 h without food or water (2 mice were placed in one chamber; Fig. 1*A*). Before and immediately after heat exposure, body weight was measured and mice were allowed to recover at 25°C and 40% relative humidity with food and water. Core temperature was also measured before, immediately after, and at 1 h after heat exposure using a rectal thermometer (AD1687, A&D Company, Tokyo, Japan). Sham mice underwent the same experimental procedures but with the temperature in the chamber maintained at 25°C (Fig. 1*A*). Urine samples were collected from some of the subject mice on *days 1* and *3* after heat exposure. After BW was measured, mice were euthanized under deep anesthesia with 4% isoflurane on *days 0* (2 h after finishing heat exposure), *1*, *3*, and *7* for the collection of blood, urine, and kidneys.

### HA Protocol

The HA protocol was a modification of a previously described protocol and consisted of mild heat exposure for 5 days (29). HA mice underwent mild heat exposure in the environmental chamber at 38°C and 40% relative humidity for 3 h with ad libitum access to food and water. Thereafter, mice were allowed to recover at room temperature until the next day. This process was repeated for 5 days. Thereafter, mice were given a 2-day rest at 25°C and 40% relative humidity with a 12:12-h light-dark cycle. After the rest days, the above-described HS protocol was conducted (HA + HS group). Urine samples were collected from some of the subject mice on *days 1* and *3* after heat exposure. HA + HS mice were euthanized under deep anesthesia using 4% isoflurane before HS and on *days 1* and *7* after HS for sample collection. HS mice received HS without the HA protocol.

### Measurement of Blood Samples

Blood samples obtained from the mouse inferior vena cava were collected in a polyethylene tube. Serum creatinine in blood samples was measured by an enzymatic method (SRL, Tokyo, Japan).

### Preparation of Kidney Single-Cell Suspensions

To obtain kidney single-cell suspensions, we modified the established protocols for collecting liver mononuclear cells (30). Kidneys were isolated from mice and cut into small pieces after saline perfusion. Samples were treated with collagenase for 40 min at 37°C while being shaken. After cells were passed through a stainless steel mesh, they were subjected to Percoll gravitational centrifugation. The leukocyte-rich layer was collected, resuspended in red blood cell lysis solution, and filtered through a 40-μm cell strainer.

### Flow Cytometric Analyses

Following incubation with Fc blocker (Cat. No. 93, eBioscience, San Diego, CA), cell suspensions were incubated with the following antibodies for 15 min at 4°C: APC-conjugated anti-CD45 antibody (Cat. No. 30-F11, eBioscience), FITC-conjugated anti-F4/80 antibody (BM8, eBioscience), FITC-conjugated anti-major histocompatibility complex class II (MHC II) antibody (Cat. No. 14-4-4S, eBioscience), PE-cyanine 5-conjugated anti-CD11b antibody (M1/70, eBioscience), PE-conjugated anti-F4/80 antibody (BM8, eBioscience), PE-conjugated anti-Ly6C antibody (HK1.4, eBioscience), PE-conjugated anti-Ly6G antibody (Cat. No. 1A8, eBioscience), PE-conjugated anti-CD80 antibody (B7-1, eBioscience), and/or PE-conjugated anti-CD206 antibody (MR6F3, eBioscience). For the detection of Hsp70 expression of macrophages, after incubation with APC-conjugated anti-CD45 antibody, PE-conjugated anti-F4/80 antibody (BM8, eBioscience), and PE cyanine 5-conjugated anti CD11b antibody, cell suspensions were permeabilized with BD Cytofix/Cytoperm (Cat. No. 51-2090KZ, BD Biosciences, San Diego, CA) followed by incubation with anti-Hsp70 antibody (Cat. No. 5A5, Abcam). The secondary antibody used was Alexa Fluor 488 donkey anti-mouse (A21202, Invitrogen, Waltham, MA) for 30 min at 22°C. The viability of cells was analyzed using a kit of annexin V (BioLegend, San Diego, CA) and 7-amino actinomycin D (7-AAD; BioLegend). After being stained, cells were analyzed using a Novocyte flow cytometer (ACEA Bioscience, San Diego, CA). Isotype control antibodies corresponding to each fluorescently conjugated antibody were used as needed.

### Kidney Histology and Immunohistochemistry

After perfusion with saline, kidneys were fixed in 4% paraformaldehyde and embedded in paraffin. Three-micrometer sections were stained with periodic acid-Schiff reagent (Cosmo Bio, Tokyo, Japan). Tubular injuries were defined as tubular necrosis, cast formation, loss of the brush border, and tubular vacuolization (31). At least four fields at ×200 magnification from each sample were scored as follows: 0, normal; 1, percentage of injury tubules ≤10%; 2, 11–25%; 3, 26–45%; 4, 46–75%; and 5: ≥75%, as previously described elsewhere (31). For the analysis of kidney fibrosis, deparaffinized sections were incubated with picrosirius red solution (Cosmo Bio) for 1 h at room temperature and washed with acetic acid water. For immunohistochemistry, following deparaffinization and dehydration, antigen retrieval was performed by heat mediation with citrate solution (pH 6). After inhibition of nonspecific binding using Blocking One (Nacalai Tesque, Kyoto, Japan) and endogenous peroxidase using Dako REAL Peroxidase-Blocking Solution (Dako, Glostrup, Denmark), sections were incubated with anticleaved caspase-3 antibody (Asp^175^, Cell Signaling Technology, Danvers, MA) at 4°C overnight. After being washed with PBS, sections were incubated with secondary antibody Histofine Simple Stain Mouse MAX-PO (Nichirei, Tokyo, Japan) at room temperature for 40 min and then stained with diaminobenzidine. For Hsp70 staining, the Histofine Mouse Stain Kit (Nichirei) was used to block the reaction to endogenous immunoglobulin. Anti-Hsp70 antibody (Cat. No. 5A5, Abcam) was used for primary antibody. For F4/80 staining, antigen retrieval was performed using protease K (Dako) and anti-F4/80 antibody (A3-1, Bio-Rad, Hercules Laboratories) was used as the primary antibody. All images were obtained using Keyence BZ-X710 (Keyence, Osaka, Japan). For picrosirius red staining sections and Hsp70 immunohistochemistry sections, at least four fields from each sample were obtained at ×200 magnifications, respectively, and the positive areas were analyzed using ImageJ-Fiji (32). Glomeruli and large vessels were excluded from the analysis of the picrosirius red-positive area. For cleaved caspase-3 immunohistochemistry sections, positive tubules were counted in four randomly selected fields at ×200 magnification.

### Immunofluorescence Microscopy

For immunofluorescence staining of proliferating cell nuclear antigen (PCNA), following deparaffinization and dehydration, antigen retrieval was performed by heat mediation with citrate solution (pH 6). After inhibition of nonspecific binding, sections were incubated with anti-PCNA antibody (PC10, Abcam) at 4°C overnight. The next day, sections were incubated with Alexa Fluor 594 GAR (R37117, Invitrogen). FITC-conjugated *Lotus tetragonolobus* lectin (LTL) antibody (Vector Laboratories) was also used for staining proximal tubules. At least four fields from each sample were obtained, and PCNA-positive nuclei were counted using ImageJ-Fiji.

For staining of macrophages, frozen sections of kidneys were cut on a cryostat at −20°C, rehydrated in PBS, and blocked with Blocking One for 1 h. Sections were then incubated with antibodies for 1 h at 37°C in the dark. The following antibodies were used: PE-conjugated anti-F4/80 antibody (BM8, eBioscience), APC-conjugated anti-CD11b antibody (M1/70, eBioscience), and FITC-conjugated LTL antibody (Vector Laboratories). After being washed, sections were mounted with PermaFluor Aqueous Mounting Medium (Richard-Allan Scientific, San Diego, CA), and images were obtained using Keyence BZ-X710.

### Determination of Kidney Injury Molecule-1 and Neutrophil Gelatinase-Associated Lipocalin Levels in Kidney Tissues or Urine

Kidney tissues were homogenized with radioimmunoprecipitation (RIPA) buffer lysis buffer (Wako, Osaka, Japan) containing 1% protease inhibitor cocktail (Nacalai Tesque), and the supernatant was used for measuring kidney injury molecule-1 (KIM-1; Mouse KIM-1 ELISA Kit, Abcam) and neutrophil gelatinase-associated lipocalin (NGAL; NGAL ELISA Kit, Enzo Life Science, New York, NY). Urinary KIM-1 levels were also measured and normalized by urine creatinine level (Dry Chem 3500 V, Fujifilum, Tokyo, Japan).

### Statistical Analyses

Results are presented as means ± SE. Differences among groups were assessed by one-way ANOVA with Turkey’s post hoc test. Differences between the two groups were assessed by a Mann–Whitney *U* test. A correlation analysis was conducted using Pearson’s correlation coefficient. All statistical analyses were performed using the JMP software program (version 15, SAS Institute, Cary, NC). *P* values of <0.05 were considered to indicate statistical significance.

## RESULTS

### HS Increased Serum Creatinine Levels and Induced Tubular Injury

Mice were exposed to HS at 40°C for 1 h (HS group; Fig. 1*A*). Their core temperature was elevated immediately after HS (before HS: 36.6 ± 0.1 and after HS: 40.3 ± 0.3°C, *P* < 0.05). The serum creatinine level on *day 0* was higher in the HS group than in the sham group (sham group: 0.10 ± 0.01 mg/dL and HS group at *day 0*: 0.21 ± 0.05 mg/dL, *P* < 0.05).

Tissue KIM-1 and NGAL levels in the kidney on *day 1* were both higher in the HS group than in the sham group (Fig. 1, *B* and *D*). The urinary KIM-1 level was increased on *days 1* and *3* (Fig. 1*C*).

**Figure 2.**
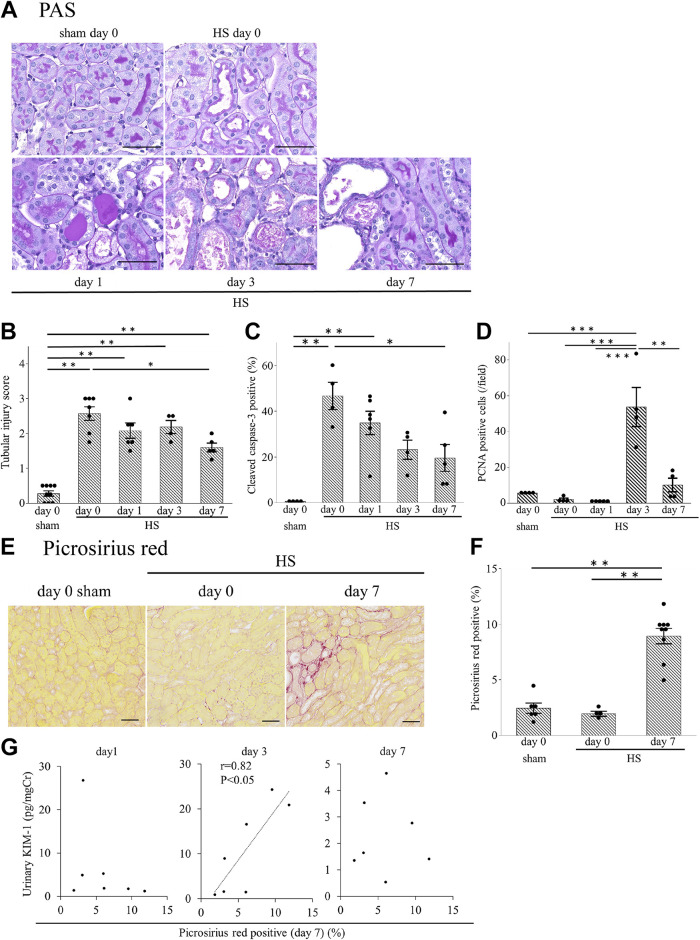
Kidney histology after heat stress (HS). *A*: representative periodic acid-Schiff (PAS) staining in the mouse kidney (magnification: ×400). *B*: the tubular injury score in the HS group was higher than that in the sham group. Tubules positive for cleaved caspase-3 (*C*) and proliferating cell nuclear antigen (PCNA; *D*) were counted in four randomly selected fields at ×200 magnification. On *days 0* and *1* after HS, the percentage of cleaved caspase-3-positive tubules in the HS group was significantly higher than in the sham group (*C*). PCNA expression in tubular cells tended to be reduced on *day 1* but was increased on *day 3* (*D*). *E*: renal fibrosis was evaluated with picrosirius red staining (magnification: ×200). *F*: the picrosirius red-positive area, as evaluated using ImageJ, was significantly higher on *day 7* compared with *day 0* in the HS group or *day 0* in the sham group. *G*: the correlation between the picrosirius red-positive area on *day 7* after HS and the urinary KIM-1 levels on *days 1*, *3*, or *7* were examined (*n* = 7). Only urinary KIM-1 on *day 3* had a significant positive correlation. Data represent means ± SE from 4 to 9 mice in each group. *P < 0.05; **P < 0.01; ***P < 0.001. Scale bars = 50 μm in *A* and *E*. Cr, creatinine.

Tubular injuries were assessed by periodic acid-Schiff staining (Fig. 2*A*). Loss of the brush border and cast formation was induced after HS on *day 0* (Supplemental Fig. S1*A*; https://doi.org/10.6084/m9.figshare.20071670.v1). The tubular injury score was significantly higher in the HS group than in the sham group (Fig. 2*B*). However, on *day 7*, the score was significantly lower than the score on *day 0* in the HS group. Mice in the HS group also showed a significantly higher percentage of cleaved caspase-3-positive tubules on *day 0* (2 h after HS) than in the sham group, and the percentage gradually decreased until *day 7* (Fig. 2*C* and Supplemental Fig. S1*B*). In the HS group, although the number of PCNA-positive tubular cells (which reflects tubular cell proliferation) on *days 0* and *1* tended to be lower compared with the sham group, positive cells increased on *day 3* (Fig. 2*D* and Supplemental Fig. S1*C*).

### Kidney Fibrosis Induced by HS

The picrosirius red-positive area, which reflects tissue fibrosis, was markedly higher on *day 7* after HS than on *day 0* (Fig. 2, *E* and *F*). Interestingly, the urinary KIM-1 level on *day 3*, but not on *days 1* or *7*, was significantly correlated with the severity of renal fibrosis on *day 7* (Fig. 2*G*).

### HS Affected the Number of Kidney Macrophages

**Figure 3.**
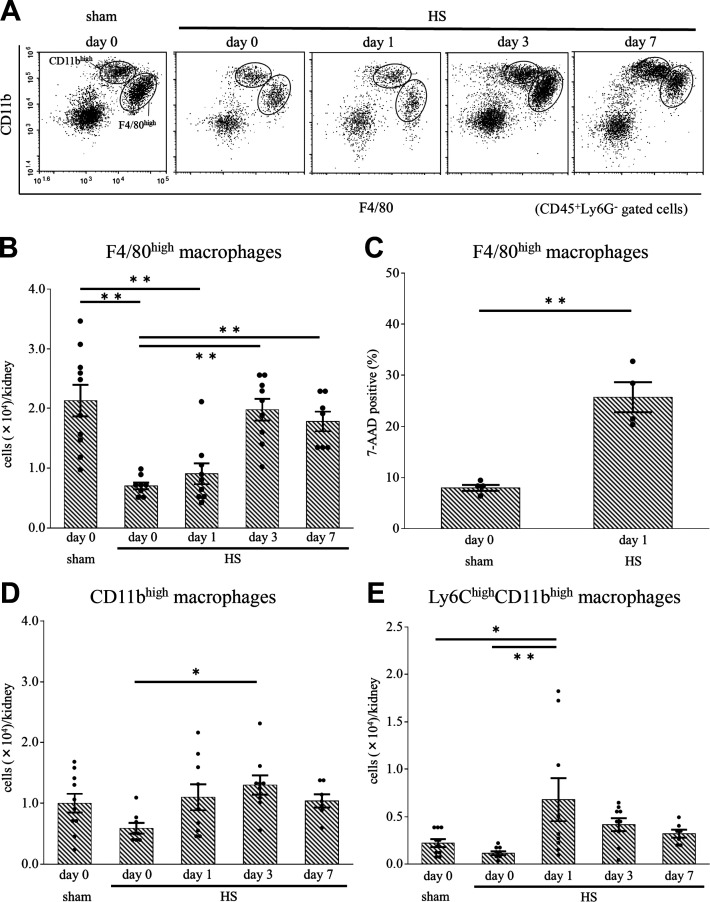
Changes in F4/80^high^ and CD11b^high^ macrophages in the kidney after heat stress (HS). *A*: using flow cytometry, CD45^+^Ly6G^−^ kidney cells from mice after HS and sham mice were classified into F4/80^high^CD11b^low^ (F4/80^high^) macrophages and F4/80^low^CD11b^high^ (CD11b^high^) macrophages. *B*: on *days 0* and *1*, the number of F4/80^high^ macrophages was significantly lower in the HS group than in the sham group. *C*: on *day 1*, the percentage of 7-amino actinomycin D (7-AAD)-positive cells (indicating nonviable cells) was significantly higher in F4/80^high^ macrophages of the HS group than in those of the sham group. *D*: the number of CD11b^high^ macrophages in the HS group did not differ from that in the sham group. On *day 1*, the number of Ly6C^high^ cells in CD11b^high^ macrophages (*E*) was significantly higher in the HS group than in the sham group. Data represent means ± SE from 8 to 10 mice in each group. *P < 0.05; **P < 0.01.

We classified kidney macrophages as F4/80^high^ CD11b^low^ (F4/80^high^ macrophages) or F4/80^low^ CD11b^high^ (CD11b^high^ macrophages) using flow cytometry (Supplemental Fig. S2) and examined the effect of HS on these kidney macrophages (Fig. 3*A*). On *days 0* and *1*, the number of F4/80^high^ macrophages in the HS group was significantly lower compared with the sham group; however, the number was restored beyond *day 3* (Fig. 3*B*). On *day 1* after HS, the percentage of 7-AAD-positive cells (indicating nonviable cells) in F4/80^high^ macrophages was markedly higher than in the sham group (Fig. 3*C*). We confirmed that these 7-AAD-positive cells were annexin V positive. A previous study has reported that kidney resident macrophages showed downregulated expression of MHC II and played important roles in tissue repair after AKI (22). In line with that study, we also observed that MHC II-negative cells appeared in F4/80^high^ macrophages after HS (Supplemental Fig. S3, *A* and *B*).

The number of CD11b^high^ macrophages did not drastically change after HS (Fig. 3*D*). However, the Ly6C^high^ population in CD11b^high^ macrophages, which are derived from bone marrow and have a proinflammatory function (18, 21), increased on *day 1* (Fig. 3*E* and Supplemental Fig. S4*A*).

Immunofluorescence experiments in which frozen kidney sections were subjected to F4/80 and CD11b staining revealed that the percentage of F4/80-positive cells on *day 1* was decreased compared with the sham group (Supplemental Fig. S5*A*), which was consistent with the result of FACS analysis. Furthermore, the addition of immunohistochemical staining of F4/80 to periodic acid-Schiff staining revealed that F4/80-positive cells were located near injured tubules on *days 3* and *7* but not on *day 1* (Supplemental Fig. S5*B*).

### HS Induced Phenotypical Changes in Kidney Macrophages

**Figure 4.**
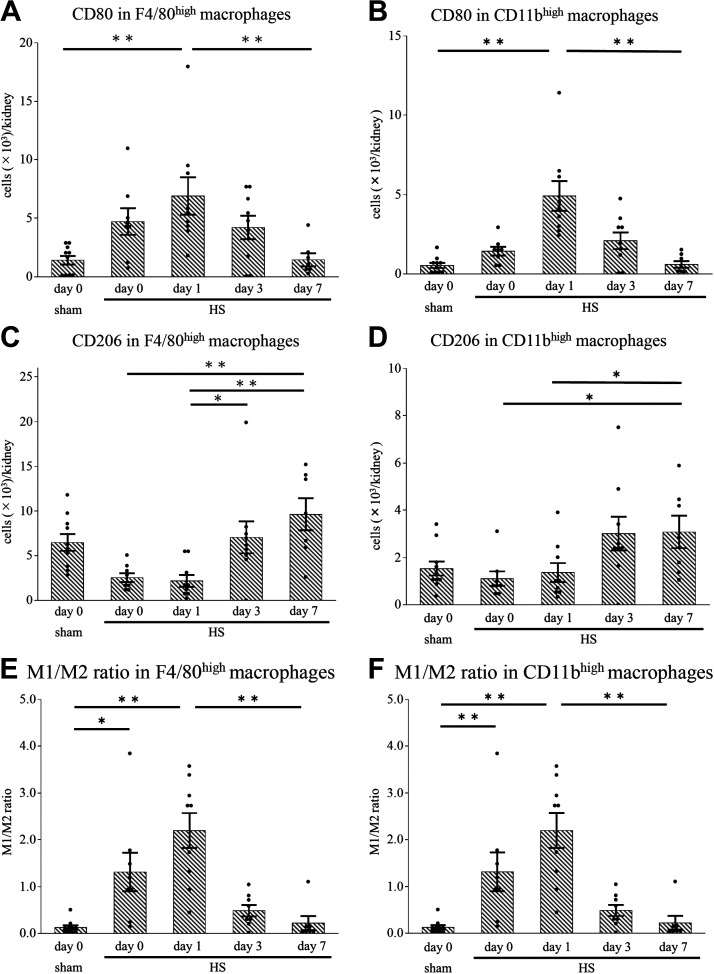
Expression of CD80 and CD206 in F4/80^high^ and CD11b^high^ kidney macrophages after heat stress (HS). Using flow cytometry, we analyzed kidney macrophages by focusing on CD80 and CD206 expression in F4/80^high^ or CD11b^high^ macrophages. On *day 1*, the population of CD80-positive F4/80^high^ macrophages (*A*) and CD11b^high^ macrophages (*B*) was larger in the HS group than in the sham group. In the HS group, the population of CD206-positive F4/80^high^ macrophages (*C*) and CD11b^high^ macrophages (*D*) on *day 7* was higher than that on *day 0*. On *day 1*, the ratio of CD80-positive to CD206-positive macrophages (the M1-to-M2 ratio) in the F4/80^high^ (*E*) and CD11b^high^ (*F*) macrophages were both significantly higher in the HS group than in the sham group, whereas in the HS group, they were significantly lower on *day 7* than on *day 1*. Data represent means ± SE from 8 to 10 mice in each group. *P < 0.05; **P < 0.01.

CD80-positive cells in both F4/80^high^ and CD11b^high^ macrophages, which are considered to be M1 macrophages, increased on *day 1* after HS but were reduced beyond *day 3* (Fig. 4, *A* and *B*, and Supplemental Fig. S4, *B* and *D*). In contrast, CD206-positive cells in both macrophage subsets, which are considered to be M2 macrophages, were increased on *day 7* compared with *days 0* and *1* (Fig. 4, *C* and *D*, and Supplemental Fig. S4, *C* and *E*). The M1-to-M2 macrophage ratio, calculated by dividing the number of CD80-positive cells by the number of CD206-positive cells, in both macrophage subsets was significantly higher on *days 0* and *1* after HS than in the sham group (Fig. 4, *E* and *F*), suggesting M1 polarization in the early phase after HS. The M1-to-M2 ratio markedly decreased on *day 7*.

### HA Prevented Steep Core Temperature Changes after HS

**Figure 5.**
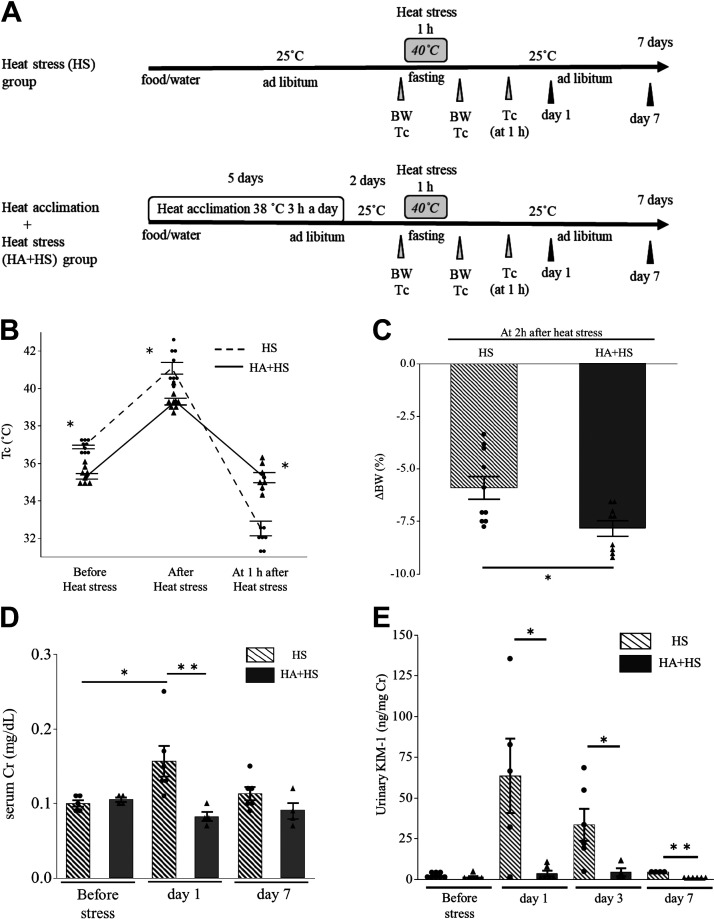
The heat acclimation (HA) protocol and changes in parameters after heat stress (HS) in the HA + HS and HS groups. *A*: mice underwent the HA protocol followed by HS (HA + HS group) and were compared with mice without HA (HS group). *B*: immediately after HS, core temperature (T_C_) was significantly lower in the HA + HS group than in the HS group; this reversed at 1 h after HS. *C*: changes in body weight (ΔBW) before and 2 h after HS were significantly higher in the HA + HS group than in the HS group. *D*: serum creatinine (Cr) did not differ between the HS and HA + HS groups before HS. However, on *day 1* after HS, serum Cr was significantly higher in the HS group than in the HA + HS group. *E*: after HS, the urinary kidney injury molecule-1 (KIM-1) level in the HA + HS group was significantly lower than in the HS group. Data represent means ± SE from 4 to 10 mice in each group. *P < 0.05; **P < 0.01.

To examine the effects of HA on HS-induced kidney injury, we made the HS group and HA + HS group (Fig. 5*A*). Compared with the HS group, the HA + HS group showed a significantly lower core temperature just before and immediately after HS. In contrast, at 1 h after HS, the HA + HS group showed a significantly higher core temperature than the HS group (Fig. 5*B*). At 2 h after HS, the HA + HS group showed a significant reduction in body weight compared with the HS group (Fig. 5*C*). These data suggest that HA + HS mice were well acclimated by accelerating heat dissipation, as previously reported (29). Before HS, serum creatinine and urinary KIM-1 did not differ according to the presence or absence of HA. However, serum creatinine levels in the HS group were significantly higher than those in the HA + HS group on *day 1* (Fig. 5*D*). Moreover, in contrast to the increase in urinary KIM-1 after HS in the HS group, urinary KIM-1 did not increase in the HA + HS group (Fig. 5*E*).

### HA Ameliorated HS-Induced Tubular Injury and Promoted Tubular Regeneration

**Figure 6.**
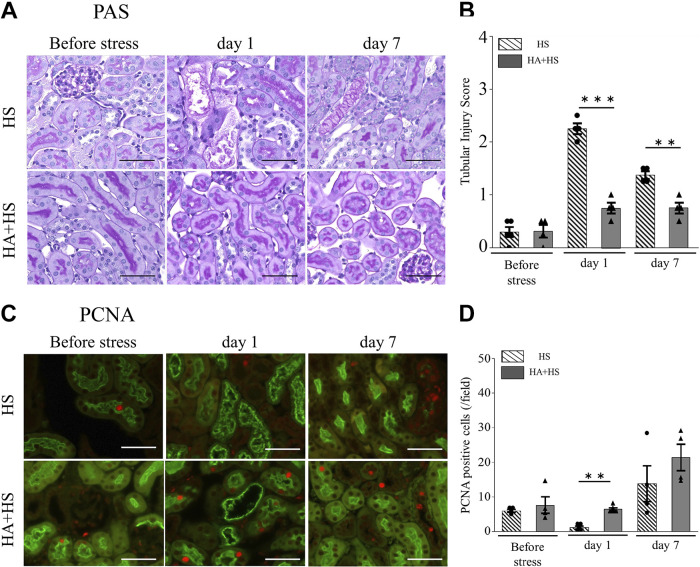
Effects of heat acclimation (HA) on kidney damage and regeneration after heat stress (HS). *A* and *C*: representative periodic acid-Schiff (PAS; *A*) and proliferating cell nuclear antigen (PCNA; *C*) stainings of kidney sections (magnification: ×400). PCNA-positive tubules were counted in four randomly selected fields. *B*: on *days 1* and *7*, tubular injury scores in the HA + HS group were significantly lower than in the HS group. *D*: the percentage of PCNA-positive tubules on *day 1* was significantly higher in the HA + HS group than in the HS group. Data represent means ± SE from 4 to 6 mice in each group. *P < 0.05; **P < 0.01; ***P < 0.001. Scale bars = 50 μm.

Before HS, the tubular injury score did not differ according to the presence or absence of HA. However, compared with the HS group, tubular injury scores in the HA + HS group were significantly lower on *days 1* and *7* (Fig. 6, *A* and *B*). On *day 1*, tubular cell proliferation, as assessed by immunofluorescence staining of PCNA, was significantly higher in the HA + HS group than in the HS group, although no difference in the number of PCNA-positive cells was observed between the HA + HS and HS groups before HS (Fig. 6, *C* and *D*).

### HA Augmented Hsp70 Expression in Tubules Before HS

**Figure 7.**
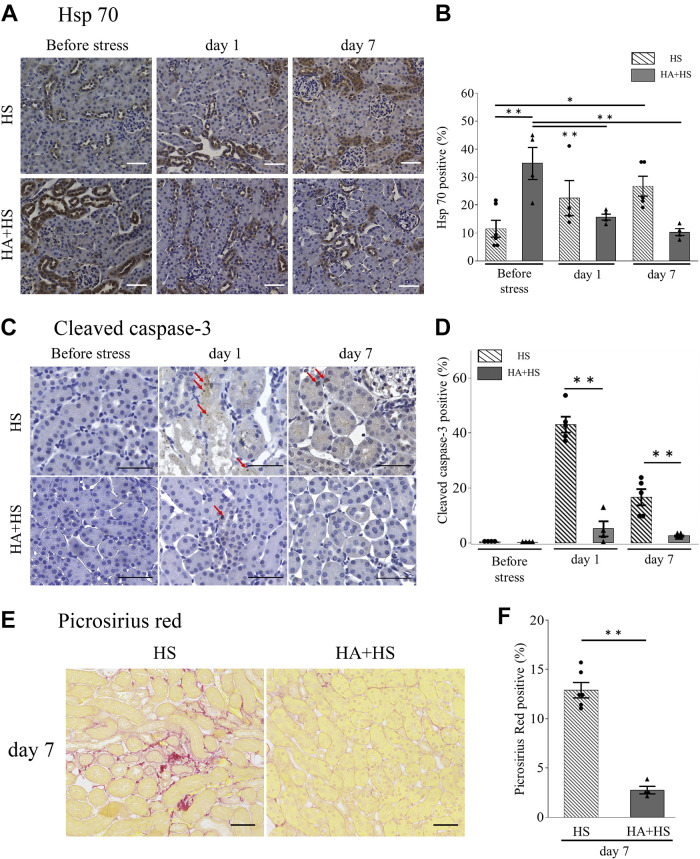
Effects of heat acclimation (HA) on kidney heat shock protein 70 (Hsp70) expression. *A*: Hsp70 staining of paraffin-embedded kidney specimens from the HA + heat stress (HS) group and HS group was compared (magnification: ×200). *B*: before HS, the percentage of the Hsp70-positive area in the HA + HS group was significantly higher than in the HS group. *C*: representative cleaved caspase-3 on paraffin-embedded kidneys from the HS and HA + HS groups (magnification: ×400). The percentage of positive tubules (red arrows) was compared. *E*: representative picrosirius red staining of kidney sections from the HS and HA + HS groups (magnification: ×200). The percentage of cleaved caspase-3-positive tubules on *day 1* (*D*) and percentage of the picrosirius red-positive area on *day 7* (*F*) were both significantly lower in the HA + HS group than in the HS group. Data represent means ± SE from 4 to 6 mice in each group. *P < 0.05; **P < 0.01. Scale bars = 50 μm.

HA induces thermal resilience by enhancing intracellular expression of Hsp70 (29). Therefore, we examined whether HA could induce intracellular expression of Hsp70 in tubular cells and reduce tubular injury after HS. Before exposure to HS, the HA + HS group showed a significantly higher percentage of Hsp70-positive tubules than the HS group (Fig. 7, *A* and *B*). In addition, on *day 1*, the percentage of caspase-3-positive tubules in the HA + HS group was significantly lower than in the HS group (Fig. 7, *C* and *D*). The percentage of Hsp70-positive tubules on *day 7* was significantly higher than that before HS in the HS group, although it decreased after HS in the HA + HS group (Fig. 7, *A* and *B*).

### HA Ameliorated Kidney Fibrosis

Next, we assessed the effect of HA on kidney fibrosis due to HS. The percentage of kidney fibrosis on *day 7* after HS, as assessed by the picrosirius red-positive area, was higher than that on *day 0* after HS in the HS group (Fig. 2, *E* and *F*). In contrast, the percentage of kidney fibrosis on *day 7* in the HA + HS group was significantly lower compared with that in the HS group (Fig. 7, *E* and *F*).

### HA Reduced HS-Induced Changes in Kidney Macrophages

**Figure 8.**
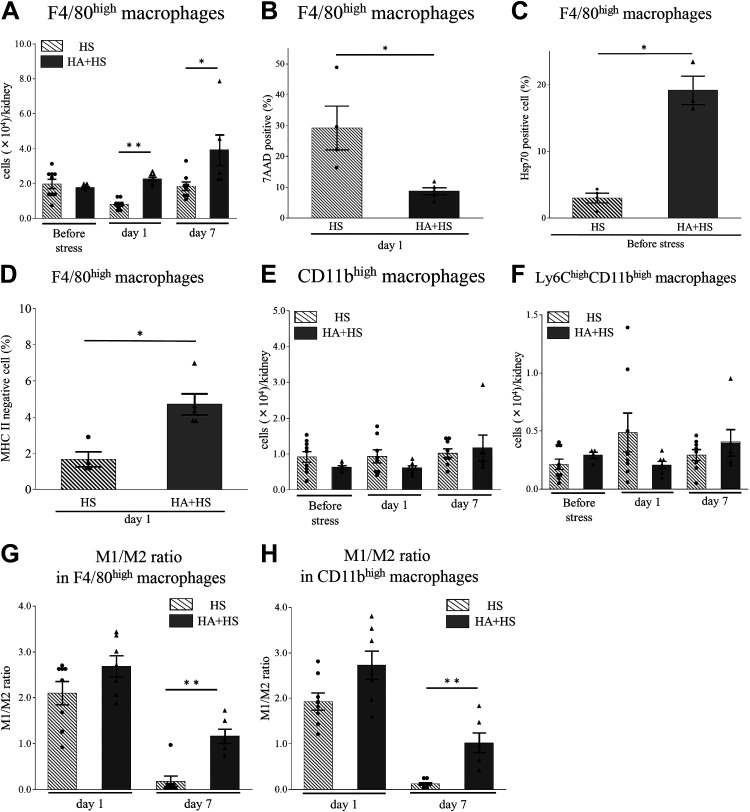
Effects of heat acclimation (HA) on changes in F4/80^high^ and CD11b^high^ kidney macrophages due to heat stress (HS). Kidney macrophages were obtained from both HA + HS and HS groups and analyzed using flow cytometry. *A*: on *days 1* and 7 after HS, the number of F4/80^high^ kidney macrophages was significantly higher in the HA + HS group than in the HS group. *B*: on *day 1* after HS, the percentage of 7-amino actinomycin D (7-AAD)-positive F4/80^high^ macrophages in the HA + HS group was significantly lower than in the HS group. *C*: before HS, the percentage of heat shock protein 70 (Hsp70)-positive cells in F4/80^high^ macrophages in the HA + HS group was significantly higher than that in the HS group. *D*: on *day 1* after HS, the percentage of major histocompatibility complex class II (MHC II)-negative cells in F4/80^high^ macrophages in the HA + HS group was significantly higher than that in the HS group. The number of CD11b^high^ macrophages (*E*) or the Ly6C^high^ subset in CD11b^high^ macrophages (*F*) before and after HS did not differ to a statistically significant extent between HA + HS and HS groups at the indicated time points. On *day 7* after HS, the M1-to-M2 ratios of F4/80^high^ kidney macrophages (*G*) and CD11b^high^ kidney macrophages (*H*) were significantly higher in the HA + HS group than in the HS group. Data represent means ± SE from 4 to 9 mice in each group. *P < 0.05; **P < 0.01.

On *day 1* after HS, mice in the HA + HS group did not show a decrease in the number of F4/80^high^ macrophages; the number in the HA + HS group was significantly higher than that in the HS group (Fig. 8*A*). On *day 1*, the percentage of 7-AAD-positive cells in F4/80^high^ macrophages was significantly lower in the HA + HS group than in the HS group (Fig. 8*B*). Before HS, the percentage of Hsp70-positive cells in F4/80^high^ macrophages in the HA + HS group was significantly higher than that in the HS group (Fig. 8*C* and Supplemental Fig. S6*A*). Moreover, on *day 1*, the percentage of MHC II-negative cells in F4/80^high^ macrophages in the HA + HS group was significantly higher than that in the HS group (Fig. 8*D* and Supplemental Fig. S6*B*). The number of CD11b^high^ or Ly6C^high^ cells in CD11b^high^ macrophages after HS did not differ to a statistically significant extent in the HA + HS group (Fig. 8, *E* and *F*). On *day 7*, the M1-to-M2 ratio in both F4/80^high^ and CD11b^high^ macrophages was significantly higher in the HA + HS group than in the HS group (Fig. 8, *G* and *H*).

## DISCUSSION

The principle finding of this study is that HA ameliorated HS-induced kidney damage. HA prevents the excessive elevation of core temperature in hot environments by accelerating heat dissipation and increasing plasma volume, which contributes to cardiovascular stability (12). Therefore, HA may have the potential to reduce kidney injury by preventing hyperthermia and dehydration (33). Nevertheless, the effects of HA remain controversial (16, 17). The present study suggests two reasons for the amelioration of kidney damage after HS by HA: the first is the increase in tubular Hsp70 expression and the second is the preservation of kidney resident macrophages, which are reduced after HS in nonacclimated mice.

In the present study, tubular expression of Hsp70 in the HA + HS group was higher than in the HS group before HS (Fig. 7*B*). Sareh et al. (29) reported that 5-day HA in mice increased expression of Hsps in the lung, heart, spleen, liver, and brain and blunted the subsequent increase in Hsp expression by HS. However, that study did not address Hsps in the kidney. Hsps mainly act as intracellular chaperones that protect the protein structure, fold under stress conditions, and are able to assist with numerous reparative processes, including the refolding of denatured proteins and removal of irreparably damaged proteins (34). Hsp70 also acts as a strong suppressor of apoptosis by inhibiting caspase-3 activation (35). Therefore, we speculate that the increase in tubular Hsp70 may have reduced cleaved caspase-3-positive tubules in the HA + HS group (Fig. 7, *B* and *D*). In line with these results, in F4/80^high^ macrophages, we also found a higher percentage of Hsp70-positive cells in the HA + HS group before HS and a lower percentage of 7-AAD-positive cells on *day 1* compared with the HS group, suggesting that HA was associated with the thermal resistance of resident macrophages (Fig. 8, *B* and *C*).

In our study, HA prevented the reduction of kidney resident F4/80^high^ macrophages on *day 1* after HS (Fig. 8*A*) and promoted tubular cell proliferation on *day 1* after HS (Fig. 6*D*). Kidney resident macrophages play important roles in the repair of kidney injury after AKI (20, 24). Several studies have demonstrated that the depletion of kidney resident macrophages inhibits recovery from AKI and exacerbates kidney fibrosis (20, 36). Furthermore, some studies have shown that AKI-responsive kidney resident macrophages, the MHC II expression of which is downregulated, are enriched with Wnt signaling and play an important role in kidney repair and development (22, 24). Consistent with these studies, our results showed that MHC II-negative cells appeared in F4/80^high^ macrophages after HS (Supplemental Fig. S3, *A* and *B*). Moreover, in the HA + HS group, we found a higher percentage of F4/80^high^MHC II-negative macrophages (Fig. 8*D*) and more PCNA-positive tubular cells on *day 1* (Fig. 6*D*) compared with the HS group. These results suggested that HA maintained F4/80^high^ kidney resident macrophages and downregulated MHC II expression of F4/80^high^ macrophages after HS, which supported tubular regeneration.

We demonstrated that not only CD11b^high^ but also F4/80^high^ macrophages exhibited the proinflammatory M1 phenotype in the early phase and the anti-inflammatory M2 phenotype in the late phase (Fig. 4, *E* and *F*). Hu et al. (37) have reported that heatstroke induced M1 macrophage infiltration in the kidney. However, they did not classify macrophages into infiltrative monocyte-derived and tissue-resident macrophages. Our results suggest that both infiltrative and resident macrophages are polarized to the M1 phenotype, due to the kidney microenvironment (38). Apoptotic cells induce the anti-inflammatory macrophage phenotype (39) and increase the promotion of tubular regeneration (40). We also showed that apoptosis of tubular cells increased in the early phase (Fig. 2*C*) and that tubular cells proliferated in the late phase in the HS group (Fig. 2*D*). Therefore, our results suggest that apoptosis of tubular cells due to HS may shift both monocyte-derived and tissue-resident macrophages to the M2 phenotype in the late phase, which may have promoted tubular regeneration. Moreover, we observed a high M1-to-M2 ratio on *day 7* in the HA + HS group (Fig. 8, *G* and *H*), which may be due to the lower percentage of apoptotic tubular cells in the HA + HS group (Fig. 7*D*).

KIM-1 is a type I transmembrane glycoprotein that is specifically induced on the apical surface of surviving proximal tubule epithelial cells after AKI. Cell-associated KIM-1 basically has an anti-inflammatory role by acting as a receptor for phosphatidylserine and mediating efferocytosis (41). However, when the ectodomain is shed, soluble KIM-1 acts as a decoy to cell-associated KIM-1 and inhibits efferocytosis (42). Therefore, the decrease in kidney tissue KIM-1 levels (presumably cell-associated) and increase in urinary KIM-1 levels (presumably soluble), which we observed on *day 3* after HS (Fig. 1, *B* and *C*), may exacerbate tubular damage. Consistently, the urinary KIM-1 level on *day 3* was positively correlated with kidney fibrosis on *day 7* (Fig. 2*G*). In line with these results, HA reduced the urinary KIM-1 level on *day 3*, with marked suppression of kidney fibrosis observed on *day 7* (Figs. 5*E* and 7*F*). Moreover, the correlation between urinary KIM-1 and kidney fibrosis implies the potential for KIM-1 to predict kidney injury. The prediction of renal sequelae in patients exposed to HS is an important issue because several studies have shown that heat-related illness can cause CKD (6, 11, 43). Further studies are needed to investigate the relationship between urinary KIM-1 and the incidence of CKD in patients with heat-related illnesses.

We conclude that AKI after HS was associated with a reduction of F4/80^high^ macrophages, which resulted in the inhibition of renal recovery from heat injury and the exacerbation of kidney fibrosis. These results suggest that heat-related illness can induce CKD. Second, HA improved kidney injury caused by HS through two mechanisms. The first is the increase in tubular intracellular Hsp expression, which may have reduced apoptosis by inhibiting caspase-3 activation. The second is the prevention of the reduction in F4/80^high^ macrophages, which augments the recovery of injured tubules. Finally, the urinary KIM-1 level on *day 3* after HS, but not on *days 1* or *7*, may have the potential to predict kidney injury. Further studies are needed to investigate the effects of HA on preventing the progression of AKI to CKD in human heat-related illnesses.

## SUPPLEMENTAL DATA

10.6084/m9.figshare.20071670.v1 Supplemental Figs. S1−S6: https://doi.org/10.6084/m9.figshare.20071670.v1.

## GRANTS

This work was supported by grants from the Ministry of Defense, Japan.

## DISCLOSURES

No conflicts of interest, financial or otherwise, are declared by the authors.

## AUTHOR CONTRIBUTIONS

H.G., M.N., and H.N. conceived and designed research; H.G., M.N., H.N., and M.N. performed experiments; H.G., M.N., and H.N. analyzed data; H.G., T.I., N.O., and M.K. interpreted results of experiments; H.G. and M.K. prepared figures; H.G. drafted manuscript; H.G., M.K., and H.K. edited and revised manuscript; H.G., M.N., H.N., M.N., T.I., N.O., M.K., and H.K. approved final version of manuscript.
